# Trends in Automated Peritoneal Dialysis Prescriptions in a Large Dialysis Organization in the United States

**DOI:** 10.2215/CJN.0000000000000436

**Published:** 2024-02-19

**Authors:** Harold E. Giles, Vidhya Parameswaran, Rachel Lasky, Linda H. Ficociello, Claudy Mullon, Dinesh K. Chatoth, Michael Kraus, Michael S. Anger

**Affiliations:** 1 Nephrology Associates PC, Birmingham, Alabama; 2 Fresenius Medical Care Birmingham Home Clinic, Birmingham, Alabama; 3 Fresenius Medical Care, Global Medical Office, Waltham, Massachusetts

**Keywords:** clinical epidemiology, dialysis, peritoneal dialysis

## Abstract

**Key Points:**

- This is the largest analysis of incident automated peritoneal dialysis (PD) prescriptions conducted in the United States to date.
- There was limited variability of automated PD prescriptions across the first 4 months of therapy.
- PD prescriptions tailored to meet the dialysis needs and lifestyle of patients may make PD a more attractive choice and increase longevity on PD.

**Background:**

Changes in health care policies and recognition of patient benefit have contributed to increases in home-based dialysis, including peritoneal dialysis (PD). Frequent monitoring and early individualization of PD prescriptions are key prerequisites for the delivery of high-quality PD. The present analysis aimed to assess variations in PD prescriptions among incident automated PD (APD) patients who remain on PD for 120+ days.

**Methods:**

This retrospective analysis examined data from patients within a large dialysis organization that initiated PD with APD between 2015 and 2019. PD prescription data were described by calendar year, timing of PD, and residual renal function categories. Changes in prescriptions from PD initiation (day 1) to day 120 were assessed descriptively.

**Results:**

The cohort included 11,659 patients. The mean age at PD initiation increased from 2015 (56 [15] years) through 2019 (58 [15] years), whereas most other variables demonstrated no clear temporal change. Most patients (86%) had nighttime PD prescribed, with an average of 4.9 (1.3) cycles per day, a mean total treatment volume of 9.3 (2.5) L, and a median daily total dwell time of 7 (6–9.5) hours. Relative to day 1 nighttime prescriptions, there were (*1*) small increases in the proportion of patients receiving three or fewer cycles per day and those receiving 6+ cycles per day, (*2*) a 100 ml mean increase in fill volume per exchange, and (*3*) a mean 0.5 L increase in total nighttime treatment volume at day 120. When changes in nighttime APD prescriptions were examined at the patient level, 49% of patients had day 120 prescriptions that were unchanged from their initial prescription.

**Conclusions:**

In the largest analysis of incident APD prescriptions conducted in the United States to date, most patients were prescribed nocturnal PD only with limited variability across the first 4 months of therapy.

## Visual Abstract

**Figure:**
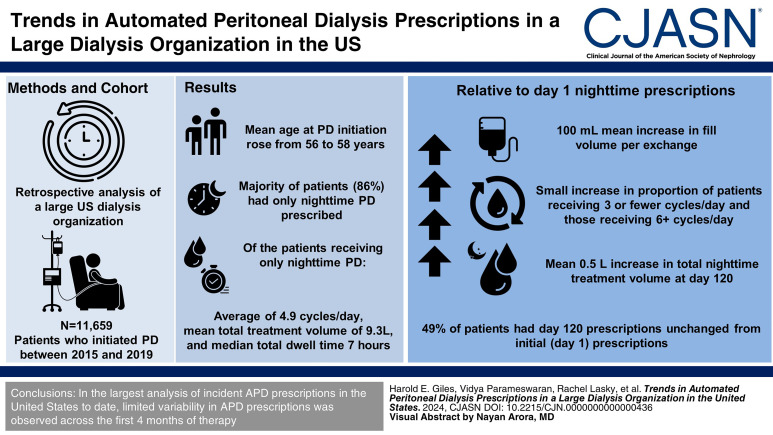

## Introduction

Peritoneal dialysis (PD) allows for the delivery of KRT in a patient's home, obviating frequent trips to, and time spent at, a dialysis center.^1^ PD is recognized as offering patients freedom, flexibility, and improved well-being combined with favorable survival, greater preservation of residual kidney function, and decreased health care costs relative to in-center hemodialysis.^2,3^ Although most (approximately 85%) patients requiring KRT qualify for PD, uptake in the United States remains low.^3^ As of 2020, 64,406 patients with prevalent kidney failure were receiving PD (12% of all dialysis among patients with kidney failure).^4^

Changes in health care policies have contributed to recent increases in home-based dialysis, including PD utilization.^3,5–7^ The adjusted total Medicare fee-for-service expenditures for beneficiaries receiving PD increased from $1.8 billion in 2010 to $2.8 billion in 2020.^4^ Although still representing a minority of KRT, the highest rates of PD utilization are observed in pediatric populations, in Asian patients, and in those with cystic kidney disease or glomerulonephritis as the underlying cause of kidney failure.^4^ Lower utilization of PD has been observed among elderly patients and many racial/ethnic minorities.^5^

The delivery of high-quality PD necessitates consideration of treatment goals that extend beyond just the adequacy of dialysis.^8,9^ Guidelines from the International Society for PD stress the importance of individualizing PD prescriptions.^8,9^ They recommend that PD prescriptions be developed after consideration of numerous factors, including functional status, social characteristics, residual kidney function, nutrition, markers of systemic peritoneal inflammation, and metabolic parameters.^8^ The prescribing clinician should also consider comorbid medical conditions, lifestyle factors, treatment adherence, and nondialytic management of kidney failure.

Changes to PD prescriptions should be made in parallel with continued assessment of clinical factors and should take into account local resources and the patient's desired lifestyle and health care goals.^8^ For instance, it has been recommended that calculation of dialysis adequacy several weeks after initiation of PD be used to guide adjustments to the prescription.^7^ It has been suggested that peritoneal membrane transport characteristics be assessed soon after initiation of PD to assist with optimization of PD prescriptions.^10^ Assessment of volume and solute removal should also be used to individualize PD prescriptions.^11^ In cases of incremental (*i.e*., low-dose) PD initiated for patients with residual kidney clearance, adjustments should be carefully tethered to reductions in residual kidney function. Changes to the PD prescription over time might include changes to the PD modality (continuous ambulatory PD [CAPD] or automated PD [APD]); adjustments to the exchange/cycle volume, frequency, and duration; modification of the PD solution; and/or changes to treatment times (*i.e*., daytime and/or nighttime).^8^

Despite expected increases in PD utilization across a more diverse group of patients, PD prescriptions in the United States have not been well characterized in the literature. An international study of PD prescriptions in a cohort of 4657 patients found marked variations across countries.^12^ That study included a contemporary evaluation of PD prescriptions in a US population (*N*=2657), but it focused on a prevalent PD population, as 62% had been on PD for more than 1 year. The present analysis aimed to describe PD prescription practices among incident APD patients.

## Methods

### Study Design

This retrospective analysis examined data from all patients who initiated PD with APD between January 1, 2015, and December 31, 2019, at any of the more than 2800 Fresenius Kidney Care (FKC) facilities across the United States. All patients were required to attend at least one training session no more than 30 days before initiating PD at home and to have received more than 120 days of PD treatment. Patients who recovered kidney function within 120 days of APD initiation and those who did not receive APD through FKC at day 120 (*e.g*., due to transplantation, death, or transfer) were excluded from the analysis. Patients with missing nighttime PD prescription data, including the number of cycles, fill volume, or dwell time, were also excluded from the present analysis. Although all patients were prescribed nocturnal APD, some patients were prescribed nocturnal APD only, and others were prescribed both daytime and nocturnal APD. Those patients with a last nighttime fill volume <500 ml and no daytime fills/cycles were assumed to have fluid for comfort only and were not considered to have had a daytime prescription.

All demographic and prescription data were deidentified and extracted from the FKC Clinical Data Warehouse. Owing to the anonymous and purely observational nature of the study, the need for informed consent was waived by an independent institutional review board (New England Institutional Review Board, Needham, MA; Work Order 17-1278966-1).

For each patient, demographic (age, sex, race, ethnicity), clinical (body surface area, body mass index [BMI], comorbidities, systolic BP), and laboratory (residual kidney function, serum phosphate, serum albumin) characteristics were extracted from electronic medical records. Dialysis vintage and the timing/duration of PD training were also recorded. The PD prescription data at day 120 was the prescription of interest; 120 days was thought to be enough time for adjustments to the initial prescription and a settled prescription to be determined. PD prescriptions at 120 days and day 1 at home (initial prescription) were extracted and included the number of cycles, fill volume per cycle, and dwell time per cycle for the nighttime and daytime. Prescription elements were described as either per cycle or per treatment day, which was shortened to per day because few patients had less than daily dialysis. Across FKC, residual kidney clearance is typically assessed *via* 24-hour urine collection with calculation of urea and creatinine clearance. At the time of the study, there was no organization-wide protocol for initial PD prescriptions, with prescriptions determined by the treating nephrologist.

### Statistical Analysis

Values for continuous variables are summarized as mean (SD), and categorical variables are presented as *No.* (%). The proportion of patients with at least one change in key APD prescription parameters at day 120 (versus day 1) was also calculated and summarized. All analyses were conducted using SAS (version 9.4; SAS Institute Inc., Cary, NC).

## Results

From 2015 through 2019, 45,468 patients began PD through FKC. Complete PD treatment, training orders, and prescription data were available for 36,974 patients. Patients who started CAPD (*n*=14,967) and those with <120 days of follow-up data (because of kidney recovery, transplant, transfer to hemodialysis, death, or loss to follow-up; *n*=10,348) were excluded. This left a total of 11,977 patients who were started on APD. Of those, 318 (3%) had missing or incomplete nighttime PD prescription data, leaving 11,659 patients in the present analysis. At PD initiation, the mean (SD) age of the overall cohort was 57 (15) years, and nearly two thirds of patients had <3 months of prior dialysis. Approximately half (51%) of the cohort was White, and most patients had a history of diabetes (55%) and/or hypertension (80%). Hyperphosphatemia (>5.5 mg/dl) and hypoalbuminemia (≤3.5 g/dl) were recorded in 43% and 38% of the cohort, respectively. Approximately 61% of the patients started PD with residual kidney function of >3 ml/min. As detailed in Table 1, the observed differences in race and ethnicity over time were largely the result of an increasing proportion of missing data. Patients were younger at the time of PD initiation in 2015 than those who initiated PD in later years (2018 and 2019). The prevalence of recorded comorbidities remained largely consistent over time, except for congestive heart failure, which decreased steadily from 8% in 2015 to 2% in 2019.

**Table 1:** Baseline demographic and clinical characteristics

Characteristic	All Years (*N*=11,659)	2015 (*n*=1546)	2016 (*n*=1921)	2017 (*n*=2438)	2018 (*n*=3150)	2019 (*n*=2604)
Age, yr, mean (SD)	57 (15)	56 (15)	55 (15)	56 (15)	57 (15)	58 (15)
Female, *No.* (%)	4821 (41)	609 (39)	796 (41)	1032 (42)	1323 (42)	1061 (41)
**Dialysis vintage, mo, mean (SD)**	17 (57)	17 (55)	15 (52)	17 (54)	17 (59)	18 (61)
≤3, *No.* (%)	7286 (63)	972 (63)	1208 (63)	1534 (63)	2007 (64)	1565 (60)
>3–6, *No.* (%)	1970 (17)	252 (16)	322 (17)	380 (16)	495 (16)	521 (20)
>6, *No.* (%)	2295 (20)	288 (19)	366 (19)	502 (21)	636 (20)	503 (19)
**Race,** *No.* **(%)**						
Black	1925 (17)	327 (21)	370 (19)	408 (17)	484 (15)	336 (13)
Other^a^	471 (4)	54 (4)	107 (6)	99 (4)	133 (4)	78 (3)
White	5911 (51)	998 (65)	1126 (59)	1380 (57)	1500 (48)	907 (35)
Missing	3352 (29)	167 (11)	318 (17)	551 (23)	1033 (33)	1283 (49)
Hispanic/Latino, *No.* (%)	1042 (9)	193 (13)	217 (11)	240 (10)	244 (8)	148 (6)
Body surface area, kg/m^2^, mean (SD)	2 (0.3)^b^	2 (0.3)	2 (0.3)	2 (0.3)	2 (0.3)	2 (0.3)
BMI, kg/m^2^, mean (SD)	31.1 (15.3)^b^	30.5 (15.0)	31.5 (15.8)	30.6 (11.6)	31.7 (17.6)	30.9 (15.1)
**Comorbidities**						
Charlson Comorbidity Index, mean (SD)	3.6 (1.4)	3.7 (1.4)	3.5 (1.4)	3.5 (1.4)	3.7 (1.4)	3.6 (1.3)
Diabetes mellitus, *No.* (%)	6409 (55)	853 (55)	1029 (54)	1259 (52)	1807 (57)	1461 (56)
Hypertension, *No.* (%)	9368 (80)	1220 (79)	1557 (81)	1962 (81)	2528 (80)	2101 (81)
Congestive heart failure, *No.* (%)	424 (4)	125 (8)	69 (4)	72 (3)	96 (3)	62 (2)
Cerebrovascular disease, *No.* (%)	484 (4)	59 (4)	73 (4)	119 (5)	139 (4)	94 (4)
Peripheral vascular disease, *No.* (%)	674 (6)	104 (7)	104 (5)	133 (6)	200 (6)	133 (5)
**Clinical and laboratory parameters**						
Systolic BP, >160 mm Hg, *No.* (%)	2943 (25)^c^	361 (23)	502 (26)	642 (26)	802 (26)	636 (24)
Residual kidney function >3 ml/min, *No.* (%)	7073 (61)	917 (59)	1175 (61)	1470 (60)	1917 (61)	1594 (61)
Serum phosphate >5.5 mg/dl, *No.* (%)	4960 (43)^d^	617 (40)	756 (39)	1050 (43)	1383 (44)	1154 (44)
Serum albumin ≤3.5 g/dl, *No.* (%)	4447 (38)^e^	560 (36)	714 (37)	945 (39)	1258 (40)	970 (37)
**PD training**						
Total training time <4 mo of initiation,^f^ d, *No.* (%)						
*≤5*	3636 (31)	532 (34)	623 (32)	746 (31)	963 (31)	772 (30)
*6–8*	4723 (41)	588 (38)	787 (41)	1004 (41)	1271 (40)	1073 (41)
*8–10*	1901 (16)	253 (16)	282 (15)	390 (16)	530 (17)	446 (17)
*>10*	1399 (12)	173 (11)	229 (12)	298 (12)	386 (12)	313 (12)
PD retraining time <4 mo of PD initiation, *No.* (%)	404 (4)	48 (3)	75 (4)	90 (4)	104 (3)	87 (3)

BMI, body mass index; PD, peritoneal dialysis.

a Other includes the following responses: American Indian/Alaskan Native, Asian, Other.

b Data missing for one patient.

c Data missing for 63 (0.5%) patients.

d Data missing for seven patients.

e Data missing for eight patients.

f Total training time includes initial training and any retraining that occurred within 4 months of initiation; for 175 (1.5%) patients, these data were missing.

Most patients (86%) had only nighttime cycles (nocturnal APD) prescribed at day 120 (Table 2). Among nocturnal APD patients, 97% were prescribed PD daily. Patients with only nocturnal APD were prescribed an average of 4.9±1.3 cycles per day, with a mean total treatment volume of 9.3±2.5 L and a median total dwell time of 7 (6–9.5) hours. Among patients receiving daytime and nocturnal cycles, 99% were prescribed PD daily. These patients had higher mean estimated dry weights, more daily cycles, larger mean total treatment volumes, and longer total dwell times than the nocturnal APD patients.

**Table 2:** Summary of day 120 peritoneal dialysis prescriptions (*N*=11,659)

Prescription Information	Nocturnal APD Patients (*n*=10,037, 86%)	Daytime+Nocturnal APD Patients (*n*=1622, 14%)
	Mean±SD or *No* (%)	Mean±SD or *No.* (%)
**Weekly frequency of PD treatments**		
≤6	232 (3)	14 (1)
7	9774 (97)	1608 (99)
Estimated dry weight, kg	83.9±21.5	90.2±23.4
Total number of cycles^a^	4.9±1.3	6.4±1.6
Total treatment volume,^b^ L	9.3±2.5	11.4±3.1
Total dwell time,^c^^,^^d^ min	420 (360–570)	1440 (555–1440)

APD, automated peritoneal dialysis; PD, peritoneal dialysis.

a Total number of cycles=nighttime cycles+daytime cycles.

b Total treatment volume=cycles×fill volume/cycle.

c Total dwell time=cycles×dwell time/cycle.

d Median (interquartile range).

**Figure 1:**
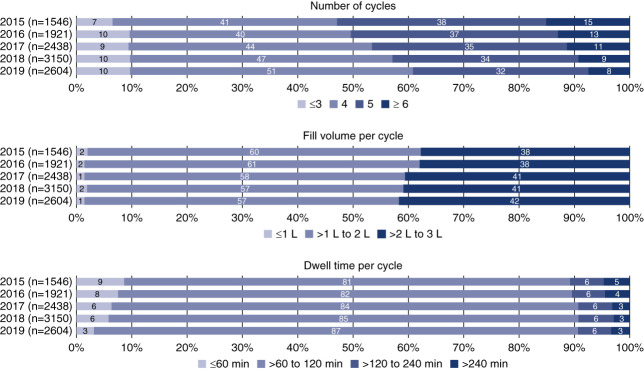
Day 120 nighttime prescriptions examined as categorical variables.

Information on 120-day nighttime prescriptions across years is shown in Figure 1. Across all years studied, the most common number of cycles per night was four, and more than 75% of patients were prescribed four or five cycles per night. The percentage of patients prescribed four cycles increased across the follow-up period (41% in 2015 and 51% in 2019), whereas the proportion of patients prescribed five cycles per night decreased (38% in 2015 to 32% in 2019). Most patients had prescribed fill volumes per cycle between 1 and 2 L, with higher fill volumes observed in later years (38% in 2015 to 42% in 2019). Dwell times of 1–2 hours per cycle were prescribed for most patients, and the percentage of patients with that dwell time increased across the follow-up period (82% in 2015 to 89% in 2019).

**Figure 2:**
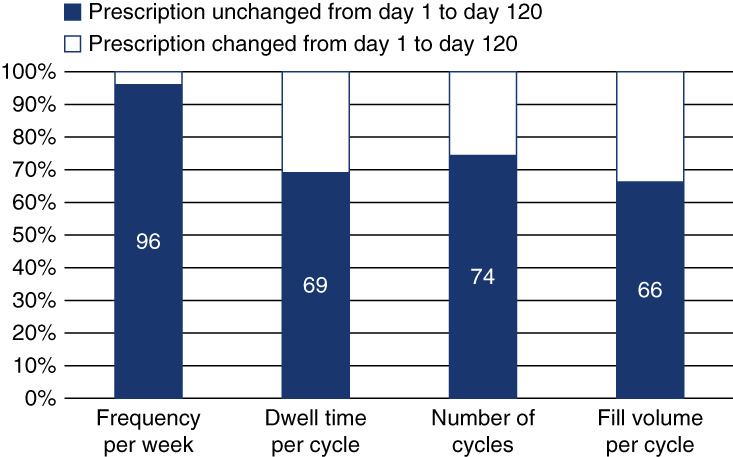
Changes in nighttime prescriptions from day 1 to day 120.

The characteristics of initial nighttime prescriptions (*i.e*., month 1) are summarized in Supplemental Figure 1. Relative to day 1 prescriptions, there were small increases in the proportion of patients receiving three or fewer cycles per day and those receiving six or more cycles per day. Fill volumes appeared to increase over the first 120 days of PD, with increases in the proportion of patients receiving more than 2 L per cycle (from 28%–32% on day 1 to 38%–42% on day 120). At day 120, dwell times per exchange remained largely unchanged from their initial values. When changes in nighttime APD prescriptions were examined at the patient level, 49% of patients had a day 120 prescription that was the same as their initial (day 1) APD prescription (Figure 2). Adjustments to 1, 2, 3, and 4 parameters occurred for 20%, 19%, 12%, and 0.5% of patients, respectively. Fill volume per cycle, the most commonly adjusted PD prescription component, was modified for 34% of patients.

There was a slight increase in the number of patients receiving daytime prescriptions at day 120 relative to day 1 (from *n*=1429 [12%] at day 1 to *n*=1622 [14%] at day 120). Among those patients not receiving daytime prescriptions at day 1 (*n*=10,406), 6% (*n*=673) had daytime prescription data at day 120. Of the 1253 patients with daytime prescription data at day 1, nearly three-quarters (*n*=933) continued to have a daytime prescription at day 120.

As summarized across Supplemental Tables 1 and 2, there were slight increases in mean number of cycles per day and fill volumes per cycle. Conversely, decreases in dwell time per cycle were observed. The proportion of patients with missing daytime prescription data was higher at day 1 than at day 120. Nighttime PD prescriptions are further summarized by year for both nocturnal APD patients and daytime and nocturnal APD patients in Table 3. Among patients receiving only nocturnal APD, there were decreases in the mean number of prescribed cycles and total treatment volume from 2015 to 2019. Among patients receiving daytime and nocturnal APD, we observed slightly decreasing mean number of prescribed cycles and no clear pattern of change in the total nighttime treatment volume or dwell time per cycle.

**Table 3:** Components of day 120 nighttime peritoneal dialysis prescription among patients receiving nocturnal automated peritoneal dialysis only and those receiving daytime and nocturnal automated peritoneal dialysis

Prescription Components	Year of PD Initiation				
	2015	2016	2017	2018	2019
Nocturnal APD					
	*n*=1338 (87%)	*n*=1675 (87%)	*n*=2077 (85%)	*n*=2659 (84%)	*n*=2288 (88%)
No. of cycles	4.6±0.9	4.6±1	4.5±0.9	4.4±0.9	4.4±0.8
Fill volume per cycle, L	2.1±0.4	2.1±0.4	2.1±0.4	2.1±0.4	2.1±0.4
Dwell time per cycle, min	90 (76–105)	90 (77–105)	90 (80–105)	90 (80–105)	90 (86–105)
Total nighttime treatment volume,^a^ L	9.6±2.5	9.4±2.5	9.5±2.7	9.3±2.7	9.2±2.5

Daytime+Nocturnal APD					
	*n*=208 (13%)	*n*=246 (13%)	*n*=361 (15%)	*n*=491 (16%)	*n*=316 (12%)
No. of cycles	4.6±1.0	4.4±1.1	4.6±0.9	4.5±1.0	4.4±0.9
Fill volume per cycle, L	2.3±0.4	2.2±0.4	2.3±0.4	2.2±0.4	2.3±0.4
Dwell time per cycle, min	91.5 (81.5–120)	97 (81–120)	90 (83–110)	95 (90–118)	95 (90–116.5)
Total nighttime treatment volume,^a^ L	10.3±2.6	9.9±2.9	10.3±2.8	9.8±2.8	10.2±2.8

APD, automated peritoneal dialysis; PD, peritoneal dialysis.

a Total nighttime treatment volume=nighttime cycles×nighttime fill volume/cycle. Data presented as mean±SD for all variables except dwell time presented in median (interquartile range).

The relationship between baseline residual kidney clearance category and components of the nighttime PD prescription is summarized in Table 4. When compared with patients with baseline residual kidney clearance ≤3 ml/min, on average, patients with >3–6 had 1.3 L less total volume, 0.8 fewer cycles, and 29 minutes shorter total dwell time, and patients with >6 had 2.4 L less total volume, 1.4 fewer cycles, and 56 minutes shorter total dwell time.

**Table 4:** Day 120 nighttime peritoneal dialysis prescriptions by residual kidney clearance at initiation

Nighttime Prescription	Residual Kidney Clearance, ml/min			
	None/Missing (Ref)	≤3	>3–6	>6
	*n*=636	*n*=3950	*n*=3970	*n*=3103
**No. of cycles**	5±1.1	4.7±0.9	4.4±0.9	4.2±0.9
≤3	23 (4)	188 (5)	318 (8)	540 (17)
4	173 (27)	1583 (40)	1975 (50)	1563 (50)
5	260 (41)	1612 (41)	1335 (34)	839 (27)
≥6	180 (28)	567 (14)	342 (9)	161 (5)
**Fill volume per cycle, L**	2.2±0.4	2.1±0.4	2±0.4	2±0.4
≤1	6 (1)	33 (1)	71 (2)	79 (3)
>1–2	260 (41)	2044 (52)	2493 (63)	1993 (64)
>2–3	370 (58)	1873 (47)	1406 (35)	1031 (33)
**Dwell time per cycle^a^, min**	90 (80–105)	90 (80–105)	90 (81–105)	90 (80–105)
≤60	36 (6)	226 (6)	245 (6)	168 (5)
>60–120	540 (85)	3416 (87)	3360 (85)	2638 (85)
>120–240	35 (6)	165 (4)	169 (4)	134 (4)
>240	25 (4)	143 (4)	196 (5)	163 (5)
Total nighttime treatment volume, L	11.3±3.1	10±2.5	9.1±2.4	8.5±2.4

Results are presented as mean±SD or *No.* (%). PD, peritoneal dialysis.

a Median presented (interquartile range).

## Discussion

This study provides insights into APD prescriptions in a contemporary cohort of incident PD patients who remained on PD for at least 120 days across a large nationwide dialysis organization. From 2015 through 2019, we documented PD being initiated in progressively older patients. Importantly, the data also indicate a wider distribution of ages at initiation, suggesting that PD is being offered to a broader group of patients.

The increased use of PD reported nationwide and the increasingly heterogeneous population receiving PD would seem to suggest an increased need to individualize PD prescriptions. Instead, our data suggest that a one-size-fits-all approach is often being taken for APD prescriptions. At the day 120 time point, nocturnal APD (*i.e*., no daytime cycles) was prescribed for most patients, with only 14% of patients receiving any daytime cycles (*i.e*., last fill volume ≥500 ml). After 4 months of PD, approximately 80% of patients received four or five nocturnal cycles of APD, and dwell times were 1–2 hours for most (>80%) patients. Similarly, more than 55% of patients were prescribed fill volumes between 1 and 2 L. It is worth noting that because of the total dwell times of nocturnal PD observed, many patients underwent cycles while they were awake. Although not available in the present dataset, if one were to include fill and drain times, it is likely that some patients were connected to the cycler for more than half of the day. Including daytime exchanges may be discussed with patients as a way to shorten the number of hours on a cycler.

Given PD physiology, changes in dialysate contact time (total dwell time) and exchange/cycle frequency can affect the removal of small and middle molecules.^13,14^ As such, variation in prescription may be needed to improve care and potentially improve outcomes across patient types. Nonetheless, changes from day 1 to day 120 were generally small. The mean total nighttime treatment volumes increased by 0.3 (2016) to 0.6 (2015) L from APD initiation to day 120. This was largely attributable to a mean 100 ml increase in fill volume per exchange. Although we report some temporal changes in PD prescriptions across years (*e.g*., small decreases in the number of cycles and total nighttime volume), the components of APD prescriptions were fairly consistent across a widening spectrum of patients.

This analysis presents unique insights into the nature of PD prescribed in the United States and builds on several previous studies examining temporal trends in PD prescriptions. In 1998, Blake and colleagues examined PD prescriptions across the United States and Canada between 1988 and 1996.^15^ During that time, use of APD increased greatly but still accounted for only one third of PD in the United States in 1996. Daytime dwells were common (70%), and nearly 10% of patients had two or more daytime cycles. In that study, patients received an average of 5.2 cycles per night (versus 4.4–4.6 in the present analysis). In addition, from 1986 to 1996, the total treatment volumes increased from 8.9 to 10.9 L/d (versus a reduction in nighttime treatment volumes in this study). Trends in US APD prescriptions from 1997 through 2003 were subsequently studied by Mujais and Childers.^16^ They observed increasing fill volumes, increasing cycler time, and reductions in the number of cycles over time. Notably, the mean total treatment volume among adults was more than 12 L during the study period.

Our findings are generally consistent with those from contemporary PD Outcomes and Practice Patterns Study (PDOPPS) APD datasets. Wang and colleagues examined variations in PD prescriptions across six countries using data from 2014 to 2017.^12^ The US population they studied was of similar age, sex, BMI, and diabetes status as the present cohort, but had longer KRT and PD vintages and higher rates of heart failure and peripheral vascular disease than the population we studied. Among the 2199 US patients receiving APD reported by Wang *et al.*, daytime exchanges were more common (50%) and nighttime dwell volumes were larger, with 64% of patients treated with more than 2 L per cycle. The other components of PD prescriptions were similar between the two studies. According to more recent (*i.e*., 2022) US data from the PDOPPS Practice Monitor, approximately 60%–65% of APD patients are treated with a total of four or five cycles per day (versus a mean [SD] of 6.2 [1.5] in 2019 in this study), approximately 40% have a daytime fill and/or exchange (defined as any fill/exchange during the day) versus 12% in 2019 in this study (defined as last nighttime fill volume ≥500 ml and daytime fill/exchange), and the mean total prescribed volume is 8 L/d (versus 9.2 L/d among nocturnal APD only patients in 2019 in this study).^17^

The present data can also be examined to highlight potential differences between practice in the United States and other countries. Consistent with US data from Wang and colleagues,^12^ we documented that most PD patients receive APD (versus CAPD). Rates of CAPD in Japan, Thailand, Australia/New Zealand, and the United Kingdom appear considerably higher.^12^ Among patients receiving APD in Thailand and the United Kingdom, nearly one third of patients receive six or more cycles a day (versus 8% [2019] to 15% [2015] in this study).^12^ The countries included in the analysis by Wang *et al.* also appear to use daytime exchanges more frequently than we observed in the United States Finally, nighttime dwell volumes >2 L, while common in our study and most countries included in the PDOPPS analysis, appear infrequent in Japan.

Although the need to individualize PD prescriptions is widely endorsed,^8^ guidance on how to adjust prescriptions on the basis of patient characteristics is limited. For instance, patients with a higher BMI and those with reduced residual kidney function should generally be treated with higher-volume therapy.^18^ Although sometimes difficult to obtain, given the associated time burden to patients and clinical staff,^19^ results of peritoneal equilibration test should also guide APD prescriptions.^20^ Patients exhibiting high solute transport are well suited for shorter dwell times and nocturnal APD, whereas those with low transport generally require longer dwell times. In this analysis, high transporters were treated with more nighttime cycles, shorter dwell times per cycle, and a marked increase in total nighttime treatment volumes.

In the United States, APD represents the most common PD modality, accounting for nearly 90% of all PD.^17^ We observed a similar pattern in our population; of the nearly 15,000 patients started on CAPD during the study period, approximately 85% were converted to APD within 120 days. Although it has not been consistently associated with lower risk of death (versus CAPD), APD allows most patients to be untethered from dialysis equipment during the day and aims to improve adherence while maintaining dialysis adequacy.^21,22^ Data have shown that APD affords patients more opportunity to participate in daytime activities, including occupational duties.^22,23^ The ability to remotely monitor real-time data offers the potential to enhance the delivery of patient care.^21,24,25^ Whether clinicians will use such systems to more frequently customize APD prescriptions has yet to be fully evaluated, but preliminary evidence suggests that such programs improve clinical outcomes and reduce health care costs.^26–28^

The results of this study should be viewed in light of several methodological limitations. In this retrospective study, only quantitative data recorded in the medical records were extracted. We have no data on potential qualitative determinants of APD prescriptions such as patient lifestyle, prior therapies/experiences, or other considerations. In addition, given the objective of this analysis, we did not examine longitudinal changes in PD prescriptions or the adequacy of dialysis achieved with each prescription. Patients with missing PD prescription data were excluded from this study, but those with other missing data were included. Furthermore, this analysis focused on APD; patients initiating (or switching to) CAPD were not included, even if they subsequently received APD. As noted above, most patients at FKC clinics started on CAPD are transitioned to APD within months. Understanding the clinical characteristics influencing the initial decision to start patients on CAPD versus APD was beyond the scope of this analysis. Importantly, the data included in this analysis represent prescription data and may not represent the KRT received by the patient because machine data were not available. Notwithstanding the above limitations, we believe that this study of nearly 12,000 patients represents the largest analysis of incident APD prescriptions conducted in the United States.

In conclusion, this large retrospective study documented slight temporal changes in APD prescriptions from 2015 through 2019 that coincided with changes in the demographics of incident APD patients. The proportion of patients receiving daytime cycles has remained low (14%), and most patients had similar nighttime prescriptions (approximately 80% of patients had 4–5 nighttime cycles, approximately 60% of patients had fill volumes between 1 and 2 L, and >80% of patients were prescribed cycles lasting 1–2 hours). These data suggest the need for additional methods to assess and personalize APD prescriptions among incident PD populations. PD prescriptions that are tailored to meet the dialysis needs and the lifestyle of patients as part of a shared decision-making process may make PD a more attractive choice for patients and increase longevity on the therapy.

## Supplementary Material

## Data Availability

Partial restrictions to the data and/or materials apply. The data underlying the findings described in this manuscript may be obtained in accordance with Fresenius Medical Care's data sharing policy. Inquiries can be made to Michael.Anger@freseniusmedicalcare.com.

## Supplemental Material

Supplemental Figure 1. Month 1 nighttime prescriptions examined as categorical variables.

Supplemental Table 1. Daytime PD prescriptions at day 1 (*N*=1429).

Supplemental Table 2. Daytime PD prescriptions at day 120.

## References

[1] Chaudhary K Sangha H Khanna R. Peritoneal dialysis first: rationale. Clin J Am Soc Nephrol. 2011;6(2):447–456. doi:10.2215/CJN.07920910 21115629

[2] Chan CT Blankestijn PJ Dember LM, . Dialysis initiation, modality choice, access, and prescription: conclusions from a kidney disease: improving global outcomes (KDIGO) controversies conference. Kidney Int. 2019;96(1):37–47. doi:10.1016/j.kint.2019.01.017 30987837

[3] Bassuner J Kowalczyk B Abdel-Aal AK. Why peritoneal dialysis is underutilized in the United States: a review of inequities. Semin Intervent Radiol. 2022;39(1):47–50. doi:10.1055/s-0041-1741080 35210732 PMC8856784

[4] US Renal Data System. 2022 USRDS Annual Data Report: Epidemiology of Kidney Disease in the United States. National Institutes of Health, National Institute of Diabetes and Digestive and Kidney Diseases; 2022. Accessed May 1, 2023. https://usrds-adr.niddk.nih.gov/2022

[5] Mehrotra R Devuyst O Davies SJ Johnson DW. The current state of peritoneal dialysis. J Am Soc Nephrol. 2016;27(11):3238–3252. doi:10.1681/ASN.2016010112 27339663 PMC5084899

[6] Li PK Chow KM Van de Luijtgaarden MWM, . Changes in the worldwide epidemiology of peritoneal dialysis. Nat Rev Nephrol. 2017;13(2):90–103. doi:10.1038/nrneph.2016.181 28029154

[7] Hansson JH Watnick S. Update on peritoneal dialysis: core curriculum 2016. Am J Kidney Dis. 2016;67(1):151–164. doi:10.1053/j.ajkd.2015.06.031 26376606

[8] Brown EA Blake PG Boudville N, . International Society for Peritoneal Dialysis practice recommendations: prescribing high-quality goal-directed peritoneal dialysis. Perit Dial Int. 2020;40(3):244–253. doi:10.1177/0896860819895364 32063219

[9] Teitelbaum I Glickman J Neu A, . KDOQI US commentary on the 2020 ISPD practice recommendations for prescribing high-quality goal-directed peritoneal dialysis. Am J Kidney Dis. 2021;77(2):157–171. doi:10.1053/j.ajkd.2020.09.010 33341315

[10] National Kidney Foundation. KDOQI clinical practice guidelines and clinical practice recommendations for 2006 updates: hemodialysis adequacy, peritoneal dialysis adequacy and vascular access. Am J Kidney Dis. 2006;48(suppl 1):S1–S322. doi:10.1053/j.ajkd.2006.05.016 17045862

[11] Akonur A Firanek CA Gellens ME Hutchcraft AM Kathuria P Sloand JA. Volume-based peritoneal dialysis prescription guide to achieve adequacy targets. Perit Dial Int. 2016;36(2):188–195. doi:10.3747/pdi.2014.00255 26293841 PMC4803365

[12] Wang AY Zhao J Bieber B, . International comparison of peritoneal dialysis prescriptions from the peritoneal dialysis outcomes and practice patterns study (PDOPPS). Perit Dial Int. 2020;40(3):310–319. doi:10.1177/0896860819895356 32063209

[13] Kim DJ Do JH Huh W Kim YG Oh HY. Dissociation between clearances of small and middle molecules in incremental peritoneal dialysis. Perit Dial Int. 2001;21(5):462–466. doi:10.1177/089686080102100506 11757829

[14] Fischbach M Zaloszyc A Schaefer B Schmitt CP. Optimizing peritoneal dialysis prescription for volume control: the importance of varying dwell time and dwell volume. Pediatr Nephrol. 2014;29(8):1321–1327. doi:10.1007/s00467-013-2573-x 23903692

[15] Blake PG Bloembergen WE Fenton SS. Changes in the demographics and prescription of peritoneal dialysis during the last decade. Am J Kidney Dis. 1998;32(6 suppl 4):S44–S51. doi:10.1016/s0272-6386(98)70161-1 9892365

[16] Mujais S Childers RW. Profiles of automated peritoneal dialysis prescriptions in the US 1997–2003. Kidney Int Suppl. 2006;70(103):S84–S90. doi:10.1038/sj.ki.5001921 17080117

[17] US-PDOPPS. Practice Monitor; 2022. Accessed January 9, 2023. https://www.dopps.org/DPM-PD/DPMSlideBrowser.aspx

[18] Negoi D Nolph KD. Automated peritoneal dialysis – indications and management. Contrib Nephrol. 2006;150:278–284. doi:10.1159/isbn.978-3-318-01347-4 16721021

[19] Gu J Bai E Ge C Winograd J Shah AD. Peritoneal equilibration testing: your questions answered. Perit Dial Int. 2023;43(5):361–373. doi:10.1177/08968608221133629 36350033

[20] Dell'Aquila R Rodighiero MP Bordoni V D'Intini V Ronco C. APD prescription: achieving the adequacy goals. Semin Dial. 2002;15(6):397–402. doi:10.1046/j.1525-139X.2002.00099.x 12437533

[21] Giuliani A Crepaldi C Milan Manani S, . Evolution of automated peritoneal dialysis machines. Contrib Nephrol. 2019;197:9–16. doi:10.1159/000496302 34569509

[22] Roumeliotis A Roumeliotis S Leivaditis K Salmas M Eleftheriadis T Liakopoulos V. APD or CAPD: one glove does not fit all. Int Urol Nephrol. 2021;53(6):1149–1160. doi:10.1007/s11255-020-02678-6 33051854 PMC7553382

[23] Julian Mauro JC Molinuevo Tobalina JA Sánchez González JC. Employment in the patient with chronic kidney disease related to renal replacement therapy. Nefrologia. 2012;32(4):439–445. doi:10.3265/Nefrologia.pre2012.Apr.11366 22806278

[24] Foo MWY Htay H. Innovations in peritoneal dialysis. Nat Rev Nephrol. 2020;16(10):548–549. doi:10.1038/s41581-020-0283-8 32300232

[25] Chaudhry RI Golper TA. Automated cyclers used in peritoneal dialysis: technical aspects for the clinician. Med Devices (Auckl). 2015;8:95–102. doi:10.2147/MDER.S51189 25653566 PMC4311757

[26] Yeter HH Manani SM Ronco C. The utility of remote patient management in peritoneal dialysis. Clin Kidney J. 2021;14(12):2483–2489. doi:10.1093/ckj/sfab111 34938532 PMC8344514

[27] Milan Manani S Baretta M Giuliani A, . Remote monitoring in peritoneal dialysis: benefits on clinical outcomes and on quality of life. J Nephrol. 2020;33(6):1301–1308. doi:10.1007/s40620-020-00812-2 32779144 PMC7416995

[28] Biebuyck GKM Neradova A de Fijter CWH Jakulj L. Impact of telehealth interventions added to peritoneal dialysis-care: a systematic review. BMC Nephrol. 2022;23(1):292. doi:10.1186/s12882-022-02869-6 35999512 PMC9396599

