# Supplementary material for: Trends in Automated Peritoneal Dialysis Prescriptions in a Large Dialysis Organization in the United States
Source: Clin J Am Soc Nephrol. 2024 Feb 19;19(6):723–31. doi: 10.2215/CJN.0000000000000436 (PMC11168828; doi:10.2215/CJN.0000000000000436)
Supplement: Supplementary file 1 [file cjasn-19-723-s001.pdf]

Supplemental Material

Supplemental Figure 1. Month 1 nighttime prescriptions examined as categorical variables.

Supplemental Table 1. Daytime PD prescriptions at day 1 (*N*=1429).

Supplemental Table 2. Daytime PD prescriptions at day 120.

Supplemental Figure 1. Month 1 nighttime prescriptions examined as categorical variables.

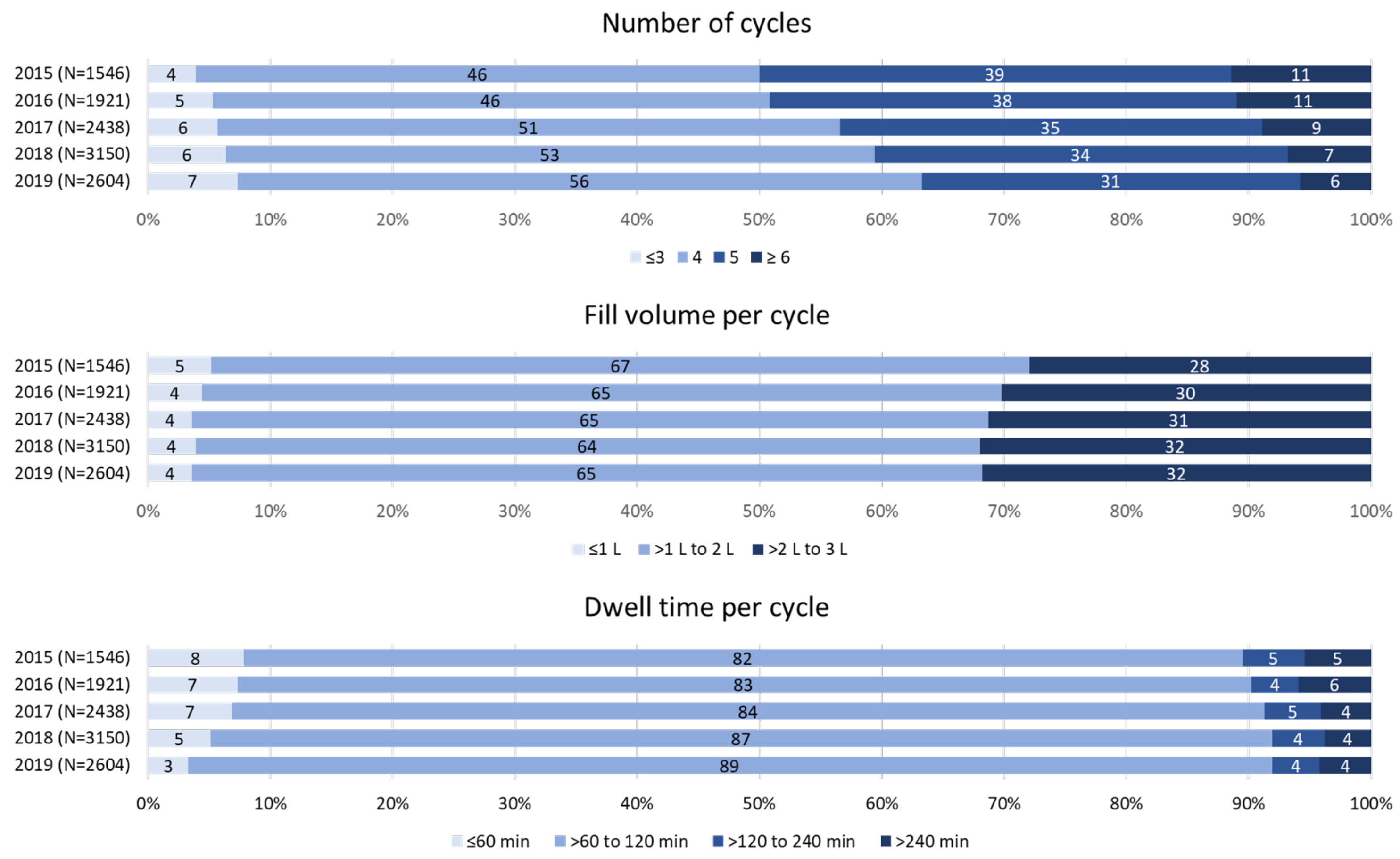

Supplemental Table 1. Daytime PD prescriptions at day 1

Daytime prescriptions	N %	Mean (SD)	Median [Q1, Q3]
Patients with daytime prescriptions[a]	1429 (12%)		
Number of cycles		0.5 (0.6)	1 [0, 1]
1	693 (49%)		
≥2	31 (2%)		
Missing	705 (49%)		
Fill volume per cycle, L		1.6 (0.8)	2 [2, 2]
≤1	78 (6%)		
>1 to 2	574 (40%)		
>2 to 4	192 (13%)		
Missing	585 (41%)		
Last fill volume, L		1.1 (0.9)	1 [0, 2]
None	560 (39%)		
>0.5 to 1	209 (15%)		
>1 to 2	538 (38%)		
>2 to 4	122 (9%)		
Dwell time per cycle, min		366.0 (135.1)	420 [240, 480]
≤120	100 (7%)		
>120 to 240	333 (23%)		
>240 to 360	199 (14%)		
>360	693 (49%)		
Missing	104 (7%)		
Total daytime treatment volume,[b] L		1.5 (1.1)	2 [0, 2]

PD, peritoneal dialysis; Q1, quartile 1; Q3, quartile 3.

[a]Patients with last fill volume <500 ml and no daytime fill or cycle were not considered to have had a daytime prescription.

[b]Total daytime treatment volume = (daytime cycles × daytime fill volume/cycle) + last fill volume.

Supplemental Table 2. Daytime PD prescriptions at day 120

Daytime prescriptions	*N* (%)	Mean ± SD	Median [Q1, Q3]
Patients with daytime prescriptions[a]	1622 (14%)		
Number of cycles		0.7 ± 0.6	1 [0, 1]
1	977 (60%)		
≥2	66 (4%)		
Missing	579 (36%)		
Fill volume per cycle, L		1.7 ± 0.8	2 [2, 2]
≤1	98 (6%)		
>1 to 2	759 (47%)		
>2 to 4	281 (17%)		
Missing	484 (30%)		
Last fill volume, L		1.1 ± 0.9	1 [0, 2]
None	618 (38%)		
>0.5 to 1	200 (12%)		
>1 to 2	608 (38%)		
>2 to 4	196 (12%)		
Dwell time per cycle, min		354.1 ± 139.3	360 [240, 480]
≤120	103 (6%)		
>120 to 240	377 (23%)		
>240 to 360	175 (11%)		
>360	597 (37%)		
Missing	370 (23%)		
Total daytime treatment volume,[b] L		1.7 ± 1.1	2 [1, 2]

PD, peritoneal dialysis; Q1, quartile 1; Q3, quartile 3.

[a]Patients with last fill volume <500 ml and no daytime fill or cycle were not considered to have had a daytime prescription.

[b]Total daytime treatment volume = (daytime cycles × daytime fill volume/cycle) + last fill volume.
